# Transmission Dynamics of Bovine Anaplasmosis in a Cattle Herd

**DOI:** 10.1155/2018/4373981

**Published:** 2018-05-02

**Authors:** Taylor A. Zabel, Folashade B. Agusto

**Affiliations:** ^1^Department of Molecular Biosciences, University of Kansas, Lawrence, KS, USA; ^2^Department of Ecology and Evolutionary Biology, University of Kansas, Lawrence, KS, USA

## Abstract

Bovine anaplasmosis is an infectious disease of cattle caused by the obligate intercellular bacterium,* Anaplasma marginale*, and it primarily occurs in tropical and subtropical regions of the world. In this study, an age-structured deterministic model for the transmission dynamics of bovine anaplasmosis was developed; the model incorporates symptomatic and asymptomatic cattle classes. Sensitivity analysis was carried out to determine the parameters with the highest impact on the reproduction number. The dominant parameters were the bovine natural and disease-induced death rates, disease progression rate in adult cattle, the mechanical devices transmission probability and contact rates, the pathogen contamination, and decay rates on the mechanical devices. The result of the sensitivity analysis suggests that control strategies to effectively prevent/control the spread of bovine anaplasmosis should focus on these parameters according to their positive or negative effect as seen from the sensitivity index. Following the results of the sensitivity analysis, three control strategies were investigated, namely, bovine-culling, safety-control, and universal. In addition to these strategies, three effectiveness levels (low, medium, and high) were considered for each control strategy using the cumulative number of newly infected cases in both juvenile and adult cattle as measure function. The universal strategy (comprising both cattle-culling and safety-control strategies) is only marginally better at reducing the number of infected cattle compare to the safety-control strategy. This result suggests that efforts should be aimed at improving and maintaining good hygiene practices; furthermore, the added benefit of culling infected cows is only minimal and not cost-efficient.

## 1. Introduction

Bovine anaplasmosis is an infectious disease of cattle caused by the obligate intercellular bacterium,* Anaplasma marginale*, which is of the order Rickettsiales [1]. The disease primarily occurs in tropical and subtropical regions and can provide significant issues regarding beef and dairy production-potential if left untreated [2]. Bovine anaplasmosis has been reported in every state within the United States, and it has been endemic in Mexico, Central America, South America, and the Caribbean Islands [3].* Anaplasma marginale* is host-specific, with only a few reported cases found in sheep and goats. It has only been found to infect erythrocytic cells in cattle under natural conditions.

The tick is considered the primary vector for this disease, and it acquires* A. marginale* by feeding on infected erythrocytes in cattle. It then acts as a reservoir by replicating in several tissues, but primarily in the midgut and salivary glands, with the latter of greater importance for transmission back to cattle [4].* A. marginale* is capable of vertical transmission in tick species, so it is quite an effective reservoir of the disease. Ticks go through four life stages: egg, larvae, nymph (juvenile), and adult. All stages of the tick's life cycle require feeding on blood, although the target of feeding usually changes at each stage. Ticks usually feed on cattle during the nymph and adult stages, which is when transmission of the disease between species occurs [5]. Vaccines have been created for* A. marginale* in cattle, but they only alleviate the symptoms and do not protect cattle from persistent infection as a carrier [6].* A. marginale* vaccine has also not yet been approved by the USDA [7].

The incubation period of the infection for cattle varies depending on the dose of the infective agent and ranges from 7 to 60 days, with an average of 28 days [2]. Signs and symptoms include fever, weight loss, abortion, and potentially death (for cattle older than 2 years), although juvenile cattle less than 9 months old are usually asymptomatic. Cattle who survive exposure to* Anaplasma* become immune to the disease; however, they carry the disease for life, which is a concern for na$\ddot{i}$ve portions of the population [7].

Before the early 1900s, anaplasmosis was commonly confused with another disease, babesiosis, which is of the genus* Babesia* in protozoa [8]. Epidemiologically, they share similar transmission paths and appear to affect cattle at a greater symptomatic intensity as they age. Ticks, biting flies, and stable flies are the most common vectors for anaplasmosis, but mechanical transmission can also occur through fomites or surgical equipment if they are not properly sterilized [2]. Mechanical transmission is likely the only agent to spread bovine anaplasmosis in regions where the aforementioned vectors are absent.

Bovine anaplasmosis is significant enough to study using a mathematical model due to its economic impact on farmers and ranchers. When* Anaplasma* infects a previously uninfected herd, the producer can expect a 3.6% reduction in successful calving, 30% increase in the cull rate, and 30% mortality in adults showing signs [14, 15]. These rates are a significant detriment to cattlemen, and preventative strategies for the disease could be better incorporated using a target reproduction number which includes accurate geographical data and disease progression. Currently, only one known mathematical model exists to determine the spread of* A. marginale*, and it was a Bayesian Space-Time model used to determine the probability of the disease based on climate [1].

The aim of this study is to develop a more general transmission compartmental model which incorporates the juvenile and adult classes of cattle, the nymph (juvenile) and adult classes of ticks, and an environmental portion to account for mechanical transmission of the disease. Also the incorporation of an asymptomatic infectious class for the adult cattle as a complement to the symptomatic infectious class which usually contains older adult cattle is noteworthy. To the best of our knowledge, this is the first compartmental model developed to study the disease transmission. Using this model, we aim to understand the basic properties of the model, such as the model stability at the disease-free equilibrium. We will also use the model to investigate the impact of different control measures on disease spread within a cattle herd following the results obtained from sensitivity analysis.

## 2. Material and Methods

### 2.1. Formulation of the Model

This model was created as follows by incorporating three subgroups: cattle, tick, and mechanical modes of transmission such as syringe needles used in the administration of antibiotics (denoted here as the environment). The cattle population is divided into juvenile (cattle less than 9 months old) and adult subpopulations, and it is further divided into susceptible (*S*_*i*_), asymptomatic infectious (*A*_*i*_), symptomatic infectious (*I*_*A*_), and carrier (*C*_*i*_) compartments, where *i* = *J*, *A* for juvenile and adult subpopulations, respectively. As previously mentioned, juvenile cattle do not experience symptoms of the disease, so they do not have asymptomatic infectious subpopulation. Therefore, the total cattle population is given as

(1) $$\begin{array}{c} N_{B}=S_{J}+A_{J}+C_{J}+S_{A}+A_{A}+I_{A}+C_{A}. \end{array}$$

The tick population was sequestered into nymph and larvae classes, each containing a susceptible class (*S*_*Ti*_) and an infectious class (*I*_*Ti*_), where *i* = *J*, *A* for nymph and larvae classes, respectively. Thus, the total tick population can be defined as

(2) $$\begin{array}{c} N_{T}=S_{TJ}+I_{TJ}+S_{TA}+I_{TA}. \end{array}$$

Individuals move between compartments according to their disease status. The susceptible (*S*_*J*_) juvenile cattle population increases via recruitment through either birth or grafting into the herd at the rate (Π_*J*_). It is assumed that vertical transmission of the disease does not occur because it has only been shown experimentally (through third-trimester exposure) and has not been shown to occur naturally [16]. Thus, for simplicity, it is assumed that any juvenile cattle coming into the herd are too young to have been exposed to the disease. Thus, there is no inflow into the asymptomatic infectious (*A*_*J*_) or carrier (*C*_*J*_) juvenile cattle classes. The juvenile population is reduced through maturation at the rate (*α*_*B*_) or through the natural death at the rate (*μ*_*B*_). The force of infection in the juvenile cattle (i.e., the rate at which the juvenile cattle are infected) is given as

(3) $$\begin{array}{c} \lambda_{J}=\frac{\beta_{J}\phi_{T}\left(I_{TJ}+I_{TA}\right)}{S_{J}+A_{J}+C_{J}}+\beta_{E}\phi_{E}E_{M}, \end{array}$$

where the parameters *β*_*J*_ and *β*_*E*_ are the probabilities that infection will occur if a juvenile cow is bitten by an infectious tick or poked by a mechanical device carrying the pathogen. The parameters *ϕ*_*T*_ and *ϕ*_*E*_ denote the tick biting rate and contact rate of the juvenile cow with a mechanical device, respectively. This assumes that each tick bite or mechanical device contact occurs at a constant rate and that this is shared among all the juvenile cattle hosts within the population. The susceptible juvenile cattle move out of the susceptible compartment at the rate (*λ*_*J*_) to the asymptomatic class. Thus, the equation for the susceptible juvenile cattle population is given as follows:

(4) $$\begin{array}{c} \frac{dS_{J}}{dt}=\Pi_{J}-\lambda_{J}S_{J}-\left(\;\alpha_{B}+\mu_{B}\;\right)S_{J}. \end{array}$$

It is assumed that juvenile cattle are not symptomatically infectious because they rarely exhibit acute symptoms [11]. As a result, death related to the disease is not incorporated into the model formulation. The population of asymptomatic infectious juvenile cattle decreases at the rate (*σ*_1_) to the carrier class. This class also maturate at the rate (*α*_*B*_) and experience natural death at the rate (*μ*_*B*_). Thus, the asymptomatic infectious compartment is given as

(5) $$\begin{array}{c} \frac{dA_{J}}{dt}=\lambda_{J}S_{J}-\left(\;\sigma_{1}+\alpha_{B}+\mu_{B}\;\right)A_{J}. \end{array}$$

The carrier class for juvenile cattle receives members of the asymptomatic infectious juvenile class and is depleted through maturation at the rate (*α*_*B*_) and natural death at the rate (*μ*_*B*_). It is assumed that juvenile cattle within this class remain carriers for the rest of their time as a juvenile and hold their carrier status into adulthood [7]. There is no anaplasmosis related death in this compartment due to carriers' immunity to the pathogen. Therefore, the equation is given as

(6) $$\begin{array}{c} \frac{dC_{J}}{dt}=\sigma_{1}A_{J}-\left(\;\alpha_{B}+\mu_{B}\;\right)C_{J}. \end{array}$$

The corresponding compartments for susceptible, asymptomatic infectious, and carrier for the adult cattle classes are similarly given, and the rate at which adult cattle acquire the infection (force of infection) is given as

(7) $$\begin{array}{c} \lambda_{A}=\frac{\beta_{A}\phi_{T}\left(I_{TJ}+I_{TA}\right)}{S_{A}+A_{A}+I_{A}+C_{A}}+\beta_{E}\phi_{E}E_{M}, \end{array}$$

where the parameters *β*_*A*_ and *β*_*E*_ are the probabilities that infection will occur if an adult cow is bitten by an infectious tick or poked by a mechanical device carrying the pathogen. The parameters *ϕ*_*T*_ and *ϕ*_*E*_ denote the tick biting rate and contact rate of the adult cow with a mechanical device, respectively. We assume that a fraction *p* of infected susceptible adult cattle move into the asymptomatic class and the remaining (1 − *p*) fraction move into the symptomatic infectious adult cattle class. The asymptomatic and symptomatic infectious adult cattle progress to the carrier class at the rates (*σ*_2_) and (*σ*_3_), respectively. It is also assumed that asymptomatic adult cattle do not die due to the infection [11]. We further assume that *σ*_1_ > *σ*_2_ > *σ*_3_, meaning that younger cattle have a faster rate of progressing from asymptomatic and symptomatic classes into the carrier class [17]. Also, it is assumed that adult cattle are much likely than juveniles to move between populations, due to their ability to be sold individually. Therefore, the adult symptomatic infectious cattle are the only compartment with disease-induced death due to anaplasmosis at the rate (*δ*). Thus, the equation for the symptomatic infectious adult cattle class is given as

(8) $$\begin{array}{c} \frac{dI_{A}}{dt}=\Pi_{A}+\left(\;1-p\;\right)\lambda_{A}S_{A}-\left(\;\sigma_{3}+\mu_{B}+\delta_{B}\;\right)I_{A}. \end{array}$$

The population of nymph (juvenile) ticks which are susceptible to anaplasmosis (*S*_*TJ*_) is generated through the recruit at the rate (*k*Π_*T*_). Ticks have been found to vertically transmit anaplasmosis to their new offspring, so *k*Π_*T*_ is the fraction of nymphs born by healthy ticks, and the remaining fraction ((1 − *k*)Π_*T*_) represents the fraction born by infectious ticks (*I*_*TJ*_). Susceptible nymph ticks move to the infectious nymph compartment at a rate (*λ*_*T*_) given as

(9) $$\begin{array}{c} \lambda_{T}=\frac{\beta_{T}\phi_{T}\left(A_{J}+C_{J}+A_{A}+C_{A}+I_{A}\right)}{N_{B}}, \end{array}$$

where the parameter *β*_*T*_ is the probability that infection will occur if a susceptible tick bites any infected or carrier cow. Both the susceptible and infectious nymphs mature into adult ticks at the rate *α*_*T*_ and are removed by natural death at the rate (*μ*_*T*_). This leads to the following system of equations for juvenile ticks:

(10) dSTJdt=kΠT−λTSTH−αT+μTSTJ,dITJdt=1−kΠT+λTSTJ−αT+μTITJ.

Adult tick susceptible and infectious equations are similarly determined.

The last compartment to be considered is the environmental portion of the model. The environmental compartment is composed of any mechanical device by which anaplasmosis can be spread; these include any unsterilized needles, scissors, knives, or anything else capable of collecting infected erythrocytes and placing them within another cattle host. Pathogen enters into the environment at a rate (*ε*) and has a decay rate due to pathogen death at the rate (*μ*_*E*_). This sets the equation for the environment as

(11) $$\begin{array}{c} \frac{dE_{M}}{dt}=\varepsilon_{E}\left(\;A_{J}+C_{J}+A_{A}+C_{A}+I_{A}\;\right)-\mu_{E}E_{M}. \end{array}$$

Given the above assumptions we have the following deterministic system of nonlinear differential equations for the transmission dynamics of bovine anaplasmosis:

(12) dSJdt=ΠJ−αBSJ−λJSJ−μBSJ,dAJdt=λJSJ−αBAJ−σ1+μBAJ,dCJdt=σ1AJ−αBCJ−μBCJ,dSAdt=ΠA+αBSJ−λASA−μBSA,dAAdt=ΠA+αBAJ+pλASA−σ2+μBAA,dIAdt=ΠA+1−pλASA−σ3+μB+δIA,dCAdt=ΠA+αBCJ+σ2AA+σ3IA−μBCA,dSTJdt=kΠT−αTSTJ−λTSTJ−μTSTJ,dITJdt=1−kΠT−αTITJ+λTITJ−μTITJ,dSTAdt=αTSTJ−λTSTA−μTSTA,dITAdt=αTITJ+λTSTA−μTITA,dEMdt=εEAJ+CJ+AA+CA+IA−μEEM.

**Figure 1:**
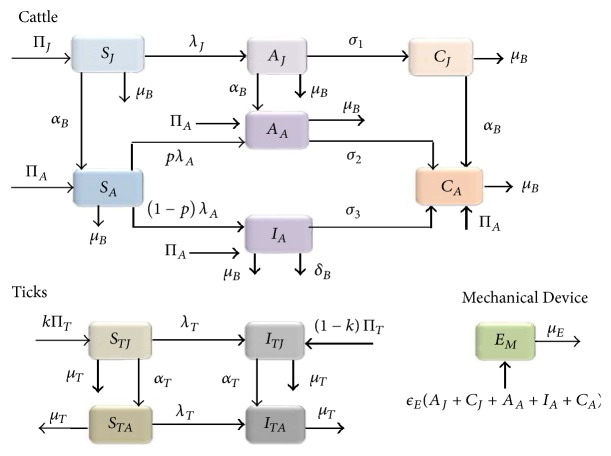
Flow diagram of the juvenile and adult cattle bovine anaplasmosis model (12). The cattle population is divided into susceptible (*S*_*i*_), asymptomatic infectious (*A*_*i*_), symptomatic infectious (*I*_*A*_), and carrier (*C*_*i*_) compartments, where *i* = *J*, *A* for juvenile and adult subpopulations, respectively. The tick population consists of susceptible (*S*_*Ti*_) and infectious (*I*_*Ti*_) classes, where *i* = *J*, *A* for nymph and larvae classes, respectively. Colors blue, purple, and orange represent susceptible, infectious, and infectious asymptomatic and carrier cattle, respectively. For ticks, tan and grey represent susceptible and infected ticks. The brighter shade represents juveniles (cattle or ticks); darker shade represents adults (cattle or ticks). The mechanical device is represented by green.

For conceptualization, a flow diagram of the bovine anaplasmosis model with juvenile and adult cattle, juvenile and adult ticks, and mechanical transmission is shown in Figure 1. The corresponding parameters and variables are described in Description of the Parameters and Variables for the Bovine Anaplasmosis Model (12).

### 2.2. Analysis of the Model

#### 2.2.1. Basic Qualitative Properties

*Positivity and Boundedness of Solutions*. For the bovine anaplasmosis transmission model (12) to be epidemiologically meaningful, it is important to prove that all its state variables are nonnegative for all time. In other words, solutions of the model system (12) with nonnegative initial data will remain nonnegative for all time *t* > 0.

**Lemma 1 .**

Let the initial data *F*(0) ≥ 0, where *F*(*t*) = (*S*_*J*_(*t*), *A*_*J*_(*t*), *C*_*J*_(*t*), *S*_*A*_(*t*), *A*_*A*_(*t*), *I*_*A*_(*t*), *C*_*A*_(*t*), *S*_*TJ*_(*t*), *I*_*TJ*_(*t*), *S*_*TA*_(*t*), *I*_*TA*_(*t*), *E*_*M*_(*t*)). Then the solutions *F*(*t*) of the bovine anaplasmosis model (12) are nonnegative for all *t* > 0. Furthermore

(13) lim supt→∞ NBt≤ΠBμB,lim supt→∞ NTt≤ΠTμT,lim supt→∞ EMt≤εEΠBμEμB,

where

(14) NBt=SJt+AJt+CJt+SAt+AAt+IAt+CAt,NTt=STJt+ITJt+STAt+ITAt.

The proof of Lemma 1 is given in Appendix A.

*Invariant Regions*. The bovine anaplasmosis model (12) will be analyzed in a biologically feasible region as follows. Consider the feasible region

(15) $$\begin{array}{c} \Omega =\Omega_{B}\cup \Omega_{T}\cup \Omega_{E}\subset R_{+}^{7}\times R_{+}^{4}\times R_{+}, \end{array}$$

with

(16) ΩB=SJt,AJt,CJt,SAt,AAt,IAt,CAt∈R+7:NBt≤ΠBμB,ΩT=STJt,ITJt,STAt,ITAt∈R+4:NTt≤ΠTμw,ΩE=EMt∈R+:EMt≤εEΠBμEμB.

**Lemma 2 .**

The region *Ω* = *Ω*_*B*_ ∪ *Ω*_*T*_ ∪ *Ω*_*E*_ ⊂ *ℝ*_+_^7^ × *ℝ*_+_^4^ × *ℝ*_+_ is positively invariant for the model (12) with nonnegative initial conditions in *ℝ*_+_^12^.

The prove of Lemma 2 is given in Appendix B.

In the next section, the conditions for the existence and stability of the equilibria of the model (12) are stated.

#### 2.2.2. Stability of Disease-Free Equilibrium (DFE)

The bovine anaplasmosis model has a disease-free equilibrium (DFE), in the absence of importation of infected adult cattle. The DFE is obtained by setting the right-hand sides of the equations in the model (12) to zero, which is given by

(17) E0=SJ∗,AJ∗,CJ∗,SA∗,AA∗,IA∗,CA∗,STJ∗,STA∗,ITJ∗,ITA∗,EM∗,

where

(18) SJ∗=ΠJαB+μB,SA∗=ΠJαB+ΠAαB+ΠJμBαB+μBμB,STJ∗=kΠTαT+μT,STA∗=kαTΠTαT+μTμT,

and all other disease states are equal to zero. Note that the model in the presence of constant inflow of infected animal does not have a disease-free equilibrium [18].

The stability of *ℰ*_0_ in the absence of importation of infected adult cattle can then be established using the next generation operator method on system (12). Taking *A*_*J*_, *C*_*J*_, *A*_*A*_, *I*_*A*_, *C*_*A*_, *I*_*TJ*_, *I*_*TA*_, and *E*_*M*_ as the infected compartments and then using the aforementioned notation, the Jacobian *F* and *V* matrices for new infectious terms and the remaining transfer terms, respectively, are defined as follows:

(19) F=00000βJϕTβJϕTβEϕESJ0000000000000pβAϕTpβAϕTpβEϕE000001−pβAϕT1−pβAϕT1−pβEϕE00000000βTϕTSTJ∗NB∗βTϕTSTJ∗NB∗βTϕTSTJ∗NB∗βTϕTSTJ∗NB∗βTϕTSTJ∗NB∗000βTϕTSTA∗NB∗βTϕTSTA∗NB∗βTϕTSTA∗NB∗βTϕTSTA∗NB∗βTϕTSTA∗NB∗00000000000,V=k10000000−σ1k2000000−αB0k300000000k400000−αB−σ2−σ3μB00000000k50000000−αTμT0−εE−εE−εE−εE−εE00μE,

where *k*_1_ = *σ*_1_ + *α*_*B*_ + *μ*_*B*_, *k*_2_ = *α*_*B*_ + *μ*_*B*_, *k*_3_ = *σ*_2_ + *μ*_*B*_, *k*_4_ = *σ*_3_ + *μ*_*B*_ + *δ*_*B*_, *k*_5_ = *α*_*T*_ + *μ*_*T*_.

Therefore, using the definition of *ℛ*_0_ = *ρ*(*FV*^−1^), the *ℛ*_0_ of the model is

(20) $$\begin{array}{c} R_{0}=\frac{1}{2}\frac{R_{E}+\sqrt{R_{E}^{2}+4\left(R_{J}+R_{A}\right)R_{T}}}{N_{B}KU}, \end{array}$$

where *ρ* is the spectral radius and

(21) RE=NBβEϕEεEk5μTSJk4αBk2μB+k2σ2+k3σ1+k3μBk2+σ1+SAk1k2k31−pμB+σ3+pk4μB+σ2,RJ=βJk4αBk2σ2+αBk3σ1+k2k3μB+k3μBσ1,RA=βAk1k21−pk3μB+1−pk3σ3+pk4μB+pk4σ2,RT=NBβTk1k2k3k4k5ϕE2μBμE2μTSTAk5+STJαT+STJμT,KU=k1k2k3k4k5μBμEμT.

The expression *ℛ*_*E*_ is the number of secondary infections in cattle due to transmission from a mechanical device. The expressions *ℛ*_*A*_ and *ℛ*_*J*_ are the number of secondary infections in adult and juvenile cattle, respectively, from one introduced infectious tick; and *ℛ*_*T*_ is the number of secondary infections in ticks from a single infectious cow. Further, using Theorem 2 in [19], the following result is established.

**Lemma 3 .**

The disease-free equilibrium (DFE) of the bovine anaplasmosis model (12) in the absence of importation of infected adult cattle is locally asymptotically stable (LAS) if *ℛ*_0_ < 1 and unstable if *ℛ*_0_ > 1.

**Figure 2:**
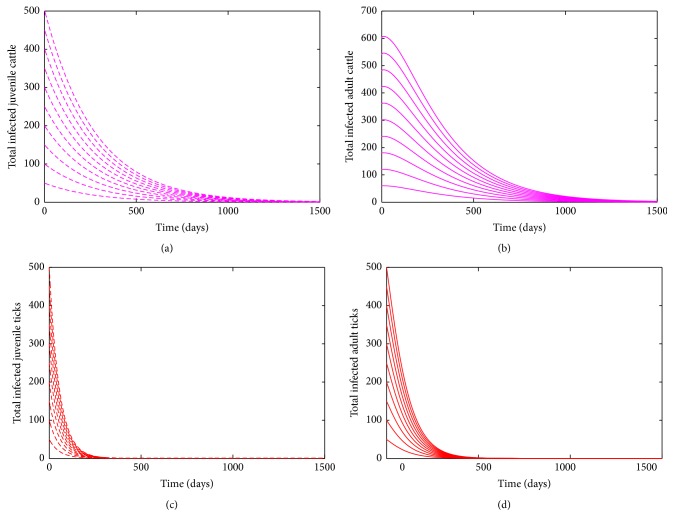
Simulation of the bovine anaplasmosis model (12) for different initial conditions as a function of time when *ℛ*_0_ < 1. The different initial conditions converges to the disease-free equilibrium when *ℛ*_0_ < 1. (a) Total number of infected (asymptomatic and carrier) juvenile cattle. (b) Total number of infected (asymptomatic and symptomatic) adult cattle. (c) Total number of infected juvenile ticks. (d) Total number of infected adult ticks. Parameter values used are as given in Table 1.

The basic reproduction number *ℛ*_0_ is defined as the average number of new infections that result from one infectious individual in a population that is fully susceptible [19–22]. The epidemiological significance of Lemma 3 is that bovine anaplasmosis will be eliminated from within a herd if the reproduction number (*ℛ*_0_) can be brought to (and maintained at) a value less than unity.  Figure 2 shows convergence of the solutions of the bovine anaplasmosis model (12) to the DFE (*ℰ*_0_) using parameter values given in Table 1 for the case when *ℛ*_0_ < 1 (in accordance with Lemma 3) with different initial conditions.

**Figure 3:**
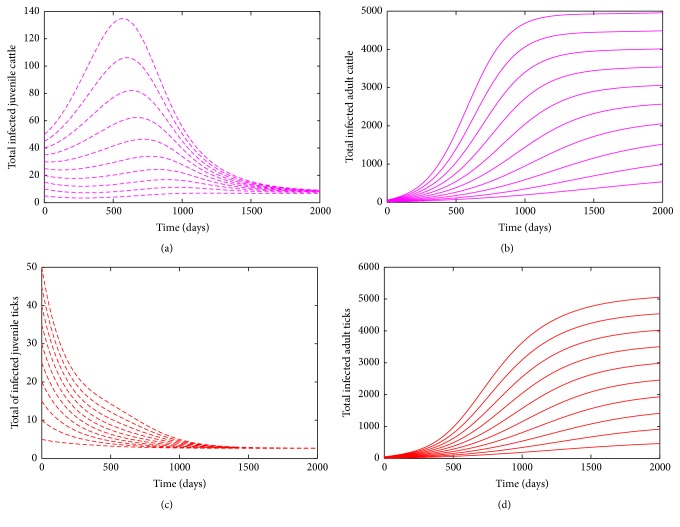
Simulation of the bovine anaplasmosis model (12) for different initial conditions as a function of time when *ℛ*_0_ > 1. The different initial conditions converges to the endemic equilibrium when *ℛ*_0_ > 1. (a) Total number of infected (asymptomatic, and carrier) juvenile cattle. (b) Total number of infected (asymptomatic, symptomatic, and carrier) adult cattle (c) Total number of infected juvenile ticks. (d) Total number of infected adult ticks. Parameter values used are as given in Table 1.

In the presence of importation of infected adult cattle, the DFE is unstable when *ℛ*_0_ > 1 [18].  Figure 3 shows convergence of the solutions of the bovine anaplasmosis model (12) to an endemic equilibrium (when *ℛ*_0_ > 1) using parameter values given in Table 1 with different initial conditions.

### 2.3. Sensitivity Analysis

In order to determine the contribution of each of the model parameters on the reproduction number, *ℛ*_0_, one can use a sensitivity analysis procedure [23–25]. Results of the sensitivity analysis help to identify the system parameters that are the best to target during an intervention and also for future surveillance data gathering. For the sensitivity analysis, a normalized forward sensitivity index is used [23–25], it determines the ratio of the relative change in *ℛ*_0_ based on a relative change in a parameter. This can be quantified if *ℛ*_0_ is differentiable by using partial derivatives such that

(22) $$\begin{array}{c} Y_{p}^{R_{0}}=\frac{\partial R_{0}}{\partial p}\times \frac{p}{R_{0}}, \end{array}$$

where *Y*_*p*_^*ℛ*_0_^ is the forward sensitivity index of *ℛ*_0_ with respect to parameter *p*; the parameter *p* is a parameter within *ℛ*_0_. The outcome of the local sensitivity analysis is shown in Table 2. The parameters with the greatest effect on *ℛ*_0_ are, therefore, those parameters with sensitivity index greater than 0.5. The parameters with the most impacts on the reproduction number, *ℛ*_0_, are the cattle (juvenile and adult) natural death rate (*μ*_*B*_), the mechanical devices contamination rate (*ε*_*B*_), the pathogen decay rate (*μ*_*E*_), the contact rate with the mechanical devices (*ϕ*_*E*_), the transmission probability per contact for mechanical device (*β*_*E*_), the disease-induced mortality rate (*δ*_*B*_), and the disease progression rate in infected adult cattle (*σ*_3_).

The results of the sensitivity analysis suggest that control strategies to effectively prevent/control the spread of bovine anaplasmosis should focus on controlling the death rate of healthy and infectious cattle through processes such as culling (*μ*_*B*_ and *δ*_*B*_, resp.); controlling the number of subcutaneous contacts (*β*_*E*_ and *ϕ*_*E*_), sterility (*μ*_*E*_), and reducing mechanical equipment contamination (*ε*_*B*_); and controlling the disease progression rate in infected adult cattle (*σ*_3_).

Therefore, control strategies which target these parameters will give the greatest impact on *ℛ*_0_. Since a 10% decrease in *ε*_*B*_, *μ*_*E*_, *ϕ*_*E*_, *β*_*E*_, and *σ*_3_, respectively, will lead to a 9.45%, 9.45%, 9.45%, 9.45%, and 6.98% reduction in *ℛ*_0_, similarly, a 10% increase in *μ*_*B*_ and *δ*_*B*_ will lead to a 19% and 8.8% decrease in *ℛ*_0_.

In the next section, we will investigate control measures that target the parameters *μ*_*B*_, *δ*_*B*_, *β*_*E*_, *ϕ*_*E*_, *ε*_*B*_, and *μ*_*E*_, respectively, with the goal of reducing the infection in the herd and eventually reducing *ℛ*_0_ less than unit.

### 2.4. Control Measures

In this section, we will investigate the impact of the dominant parameters (*μ*_*B*_, *δ*_*B*_, *β*_*E*_, *ϕ*_*E*_, *ε*_*B*_, and *μ*_*E*_) obtained from the sensitivity analysis in order to reduce the number of infected cattle in the herd.

The first two parameters (*μ*_*B*_, *δ*_*B*_) correspond to the culling of the cattle (both juvenile and adult) due to natural and disease-related deaths, increasing the values of these parameters impact the herd by removing the cattle carrying the pathogen from the herd. This effect can be captured through either culling of a diseased animal or selling off cattle within the herd. We have assumed that when such sale of diseased animal occurs, the farmers are not aware of the disease status of the animals as is the case in most farms [26].

**Figure 4:**
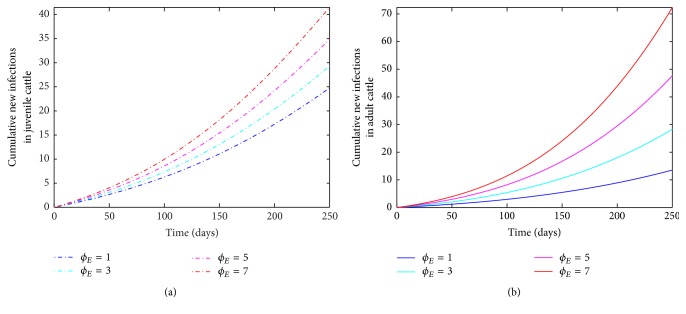
Simulation of the bovine anaplasmosis model (12) as a function of time while varying *ϕ*_*E*_. (a) Cumulative new infections in juvenile cattle; (b) cumulative new infections in adult cattle.

The next two parameters (*β*_*E*_, *ϕ*_*E*_) correspond to diseases transmission probability and subcutaneous contacts with a mechanical device with cattle. Subcutaneous contacts with mechanical device usually occur during annual vaccinations/shots; thus, increased vaccination implies an increase in the probability of disease transmission.  Figure 4 shows an increase in the number of infected juvenile and adult cattle as *ϕ*_*E*_ increases with the annual vaccination. Thus, few cases are observed when the cattle in the herd are given only one vaccination shot (this corresponds to *ϕ*_*E*_ = 1 annually), in contrast to when the herd is given seven shots annually.

**Figure 5:**
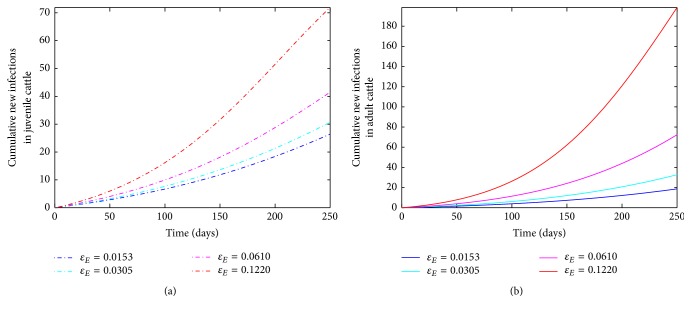
Simulation of the bovine anaplasmosis model (12) as a function of time while varying *ε*_*E*_. (a) Cumulative new infections in juvenile cattle; (b) cumulative new infections in adult cattle.

The last two parameters (*ε*_*B*_, *μ*_*E*_) are related to the mechanical devices; they represent the contamination and decay of the pathogen on the mechanical devices. As pointed above, annual vaccination shots increase subcutaneous contact with the mechanical devices such as injection, thereby increasing the contamination with pathogen these devices.  Figure 5 shows that as the contamination rate (*ε*_*B*_) increases, the number of infected animals (juvenile and adult cattle) increases.

For the rest of the section, we will investigate the effects of three control strategies, namely, the following:

- A bovine-culling strategy
- A safety-control strategy
- A combination of both strategies (universal strategy).

To reduce the number of infected animals (juvenile and adult) in the herd, additionally, three effectiveness levels (low, moderate, and high) will be considered for each of these strategies using the initial conditions given in Table 3 from [27] with some minor adaptations. By reducing the number of cattle with the disease and the possibility of interacting with the pathogen on a mechanical device, it can be implicitly inferred that the progression of disease within a population will be reduced.

The total number of susceptible individuals in the population was a total of 528 cattle and 502 ticks, with the susceptible populations being subdivided into adult and juvenile classes. The distribution of disease within the population was estimated such that susceptible cattle and ticks made up 98% of their populations and there were no cattle in the carrier class; this was done to simulate the introduction of new cattle (with ticks attached) who just recently obtained the disease and were being introduced into their new herd. The high and low effectiveness control strategy levels were set at 50 percent increase or decrease from the baseline values of these variables (*β*_*E*_, *ϕ*_*E*_, *ε*_*B*_, and *μ*_*E*_) based on their effect in lowering or increasing the number of infected cattle within the herd at a given point in time. It should be noted that these values are purely arbitrary and are only being used to theoretically determine the effect of each of these control strategies on reducing the number of infected cattle within the herd.

#### 2.4.1. Bovine-Culling Strategy

Maintaining an infection-free herd is the most effective way of controlling anaplasmosis particularly in nonendemic places [28]. A number of tests exist (serological and nucleic-acid-based tests) that would aid the detection and removal (culling) of symptomatically and persistently infected cattle from the herd [29].

Serological tests include a competitive enzyme-linked immunosorbent assay (C-ELISA), card agglutination, and complement fixation test (CFT). C-ELISA has good sensitivity and the best specificity in detecting carrier animals [29]. Due to variable sensitivity, the CFT is no longer considered a reliable test in certifying the individual disease animals [30]. Cross-reactivity between* Anaplasma* spp. can complicate interpretation of serological tests [30].

Nucleic-acid-based tests, on the other hand, have been used experimentally and are capable of detecting the presence of low level infection in carrier cattle and tick vectors [30].

Therefore, bovine anaplasmosis can be controlled by increasing the culling rate particularly of diseased animals who have failed diagnostic test for detecting* Anaplasma* within the herd. This will indirectly increase *μ*_*B*_ and *δ*_*B*_. For simulation purposes, the following three culling levels of the bovine-culling strategy considered are as follows:

1. Low culling rate of the bovine-culling strategy: *μ*_*B*_ = 8.216 × 10^−7^, *δ*_*B*_ = 3.90 × 10^−4^.
2. Moderate culling rate of the bovine-culling strategy: *μ*_*B*_ = 1.6432 × 10^−6^, *δ*_*B*_ = 7.80 × 10^−4^.
3. High culling rate of the bovine-culling strategy: *μ*_*B*_ = 3.2864 × 10^−6^, *δ*_*B*_ = 0.0016.

**Figure 6:**
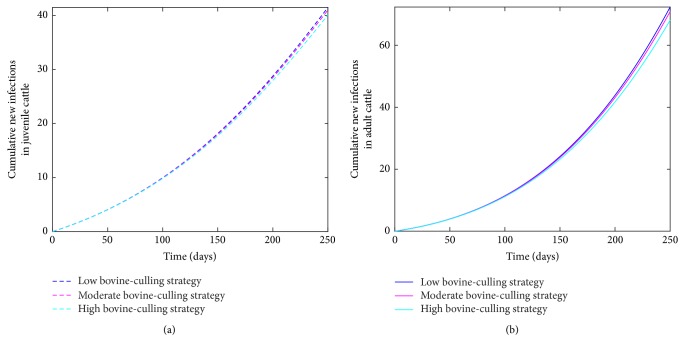
Simulation of the bovine anaplasmosis model (12) as a function of time using the bovine-culling strategy. (a) Cumulative new infections in juvenile cattle; (b) cumulative new infections in adult cattle.

Using the cumulative number of new cases of infected cattle (juvenile and adult) as evaluation measure, the model (12) was simulated for the three effectiveness levels of this strategy (see Figure 6). Comparing the outcome of these three effectiveness levels at *t* = 250 days (the end of simulation period) shows that the high bovine-culling strategy leads to a reduction in the number of new cases, this is followed by the moderate level, and the low level produces the most number of new cases, although the differences in the outcome of these strategies are negligible (see Table 4). Thus, there is a decrease in the cumulative number of new cases with increasing effectiveness level, although this strategy does not appear to have a substantial effect on reducing the disease within the herd. It should be noted that there is a lot of uncertainty inherent in the model that a difference of less than five animals for adults and one for juvenile should not be considered different.

#### 2.4.2. Safety-Control Strategy

Next, we investigate the effect of the safety-control strategy. This strategy can be accomplished through methods such as changing of needles between animals while administering vaccines or antibiotics and reducing the number of contacts the cattle have with needles, that is, adjusting the parameters *β*_*E*_, *ϕ*_*E*_, *ε*_*E*_, and *μ*_*E*_ to reflect the three control levels (low, moderate, and high) of the strategy as follows:

1. Low effectiveness of the safety-control strategy: *β*_*E*_ = 0.0015, *ϕ*_*E*_ = 0.0192, *ε*_*E*_ = 0.0610, *μ*_*E*_ = 0.50
2. Medium effectiveness of the safety-control strategy: *β*_*E*_ = 7.50 × 10^−4^, *ϕ*_*E*_ = 0.0384, *ε*_*E*_ = 0.0305, *μ*_*E*_ = 1.0
3. High effectiveness of the safety-control strategy: *β*_*E*_ = 3.75 × 10^−4^, *ϕ*_*E*_ = 0.0767, *ε*_*E*_ = 0.0153, *μ*_*E*_ = 2.0

**Figure 7:**
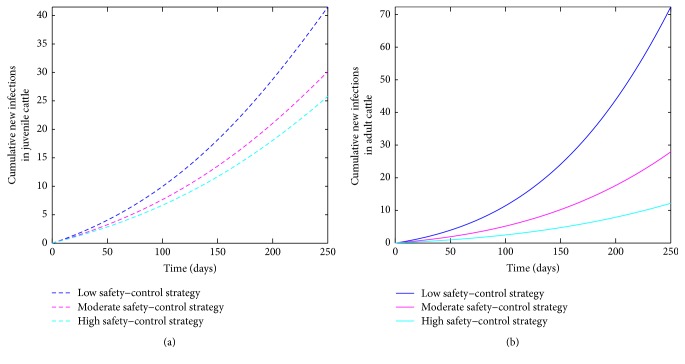
Simulation of the bovine anaplasmosis model (12) as a function of time using the safety-control strategy. (a) Cumulative new infections in juvenile cattle; (b) cumulative new infections in adult cattle.

Simulations of the model (12) show a decrease in the cumulative number of new cases with increasing levels of effectiveness (see Figure 7). The high safety-control strategy at *t* = 250 days (the end of simulation period) leads to a considerable reduction in the number of new cases compared to the moderate level (see Table 5) at the same time period. The low level performed the poorest producing the most number of new cases.

#### 2.4.3. Universal-Control Strategy

The universal-control strategy combines the cattle-culling and safety-control strategies with low to high control levels. This strategy was assessed using the following levels and parameter values:

1. Low effectiveness of the universal strategy: *μ*_*B*_ = 8.2160 × 10^−7^, *δ*_*B*_ = 3.90 × 10^−4^, *β*_*E*_ = 0.0015, *ϕ*_*E*_ = 0.0192, *ε*_*E*_ = 0.0610, *μ*_*E*_ = 0.5.
2. Medium effectiveness of the universal strategy: *μ*_*B*_ = 1.6432 × 10^−6^, *δ*_*B*_ = 7.80 × 10^−4^, *β*_*E*_ = 7.50 × 10^−4^, *ϕ*_*E*_ = 0.0384, *ε*_*E*_ = 0.0305, *μ*_*E*_ = 1.0.
3. High effectiveness of the universal strategy: *μ*_*B*_ = 3.2864 × 10^−6^, *δ*_*B*_ = 0.0016, *β*_*E*_ = 3.750 × 10^−4^, *ϕ*_*E*_ = 0.0767, *ε*_*E*_ = 0.0153, *μ*_*E*_ = 2.0.

**Figure 8:**
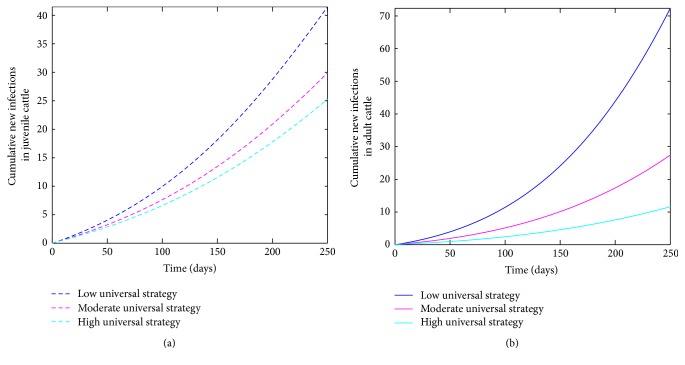
Simulation of the bovine anaplasmosis model (12) as a function of time using the universal-control strategy. (a) Cumulative new infections in juvenile cattle; (b) cumulative new infections in adult cattle.

The cumulative number of new cases of infections (juveniles and adults) is simulated for the three levels of this control strategy (see Figure 8). Comparing the three levels in Table 6 at *t* = 250 days shows that the high level leads to a considerable reduction in the number of new cases; this is followed by the moderate level and then the low level which produced the most number of new cases. Thus, this strategy provides the best control strategy aiming at eliminating bovine anaplasmosis from a cattle herd.

A comparison of the various high effectiveness levels of the three control strategies (bovine-culling control, safety control, and universal strategies) within a cattle herd at *t* = 250 days shows as expected that the universal strategy is more effective than the other two strategies implemented separately. This is followed by a safety-control strategy which is more effective than the bovine-culling control strategy in reducing anaplasmosis burden within a cattle herd. Hence, proper culling/removal of cattle coupled with diligently changing needles and sterilizing equipment and only using them when it is absolutely necessary can effectively decrease the number of infected cattle within the herd.

## 3. Discussion and Conclusion

### 3.1. Discussion

Anaplasmosis, caused by* Anaplasma marginale*, is a prevalent tick-borne disease transmitted by Rickettsia to cattle worldwide [29]. It has grave socioeconomic consequences often leading to trade restrictions both locally and internationally [29]. The costs of a clinical case of anaplasmosis in the United States on average is estimated to be over $400 per animal [14]. The effects of anaplasmosis infections on a previously uninfected herd can lead to 3.6% reduction in calf crop, 30% increase in cull rate, and 30% of adult cattle showing signs that the disease will die [14, 15]. Other economic losses include decreased milk production, severe weight loss, and poor reproductive ability [31].

In this paper, we developed and analyzed a novel model for the disease transmission dynamics of bovine anaplasmosis (to the best of our knowledge this is the first compartmental model for the disease). Notable features of the model include the incorporation of age both for ticks and cattle. Cattle less than nine months were considered juvenile cattle, and, similarly, nymph ticks were also considered as juvenile ticks. The adult classes of cattle were further stratified by whether or not they displayed symptoms of the disease; those who did were capable of dying from the disease were usually more mature cattle (older than two years) within their respective herds, and they experienced more acute symptoms due to later-life exposure to the disease.

*A. marginale* transmission typically occurs via two different routes, the biological pathway through mostly ticks and mechanical pathway [2, 13, 32]. Mechanical transmission can occur through reusing of needles, dehorners, ear taggers, castrating knives or other surgical instruments, and tattoo instruments [32, 33].

In order to determine the best methods of curtailing or eliminating the spread of anaplasmosis within a herd, a sensitivity analysis was conducted using the reproduction number (*ℛ*_0_). The parameters with the highest impact on *ℛ*_0_ are the bovine natural and disease-induced death rates (*μ*_*B*_ and *δ*_*B*_), disease progression rate in adult cattle (*σ*_3_), the mechanical devices transmission probability and contact rates (*β*_*E*_ and *ϕ*_*E*_), the pathogen contamination, and decay rates on the mechanical devices (*ε*_*B*_ and *μ*_*E*_). Knowing the effect of each of these parameters on the spread of the disease, along with incorporating control strategies which are economically and biologically feasible, will be crucial to limiting the spread of bovine anaplasmosis within a herd.

Thus, the results of the sensitivity analysis suggest that control strategies to effectively prevent/control the spread of bovine anaplasmosis should focus on controlling the death rate of healthy and infectious cattle through processes such as culling (increasing *μ*_*B*_ and *δ*_*B*_, resp.); controlling the number of subcutaneous contacts (decreasing *β*_*E*_ and *ϕ*_*E*_), sterilizing the mechanical devices (increasing *μ*_*E*_), and reducing mechanical equipment contamination (decreasing *ε*_*B*_); and, controlling the disease progression rate in infected adult cattle (decreasing *σ*_3_).

Following the results of the sensitivity analysis, three control strategies were investigated, namely, bovine-culling, safety-control, and universal. In addition to these strategies, three effectiveness levels (low, medium, and high) were considered for each control strategy using the cumulative number of newly infected cases in both juvenile and adult cattle as measure function.

All three strategies showed a reduction as the effectiveness level increases. The universal strategy (composed of both the bovine-culling and safety-control strategies) was found to be the most effective of the three strategies in terms of decreasing the cumulative number of new cases of bovine anaplasmosis. This was followed by the safety-control strategy, which was much more effective than the bovine-culling strategy in limiting the spread of anaplasmosis.

It should be noted that the universal strategy is only marginally better at reducing the number of infected cattle compare to the safety-control strategy. This implies that the efforts should be aimed at improving and maintaining good hygiene practices; furthermore, the added benefit of culling infected cows is only minimal and not cost-efficient.

These results indicate that best practices for disease control should include sterilizing (disposing of) any mechanical devices (such as syringes) that come in contact with cattle blood after each animal. “A quick rinse in a bucket of disinfectant is all that is needed” [13]. It has been shown that six out of the next ten animals could be infected if injected with a syringe used on an infected cattle [32, 33]. Practices such as culling cattle is also an effective strategy to control the infection within a herd. Cattle that survive severe anaplasmosis infection without any form of treatment do not fully recover but remain immune carriers for life and are often culled due to poor productivity [34]. Furthermore, these cattle if not removed serve as a reservoir and source of new infection to susceptible naive cattle within the herd [2]. In 2002 over 300 head of cattle were culled in Switzerland due to* A. marginale* infection [35].

Sale of infected cattle, particularly the asymptomatic ones, although may seem effective in controlling the infection within a herd, it is not advisable, as there are federal regulations governing the interstate movement of infected animals [13]. Furthermore, great care should be taken to reduce risk of disease when introducing replacement cattle into an existing herd [31]. Extra precautionary steps should be taken when introducing cattle from disease prevalent regions into a herd in a disease-free area.

It should be noted that this study did not consider the economic implication of the disease and the economic consequences of implementing the control measures. Since cattle are usually raised for their dairy or beef production, the economic consideration of the disease is a vital next step to simulate the financial impact of an anaplasmosis outbreak within a farmer's or rancher's herd and to determine the best route to reduce mortality and financial losses. Hence, the next step will be to refine the current model to more accurately follow the etiology of bovine anaplasmosis (by including a compartment for cattle treated with pharmaceuticals such as oxytetracycline) and to include a cost-effectiveness analysis for any future efforts at tackling a bovine anaplasmosis outbreak.

### 3.2. Conclusion

In conclusion, we have presented a deterministic model of a system of ordinary differential equations of anaplasmosis transmission dynamics in a cattle herd. The following results were observed from our analysis and numerical simulations:

- The model has a DFE that is locally asymptotically stable if *ℛ*_0_ < 1.
- The sensitivity analysis of the model shows that the dominant parameters on the reproduction number, *ℛ*_0_ are the cattle (juvenile and adult) natural death rate (*μ*_*B*_), the mechanical devices contamination rate (*ε*_*B*_), the pathogen decay rate (*μ*_*E*_), the contact rate with the mechanical devices (*ϕ*_*E*_), the transmission probability per contact for mechanical device (*β*_*E*_), the disease-induced mortality rate (*δ*_*B*_), and the disease progression rate in infected adult cattle (*σ*_3_).
- Numerical simulations indicate that the safety-control strategy is more effective than bovine-culling strategy, while the universal strategy is the most effective strategy for reducing anaplasmosis disease burden in a cattle herd.
- Our numerical result further suggests that more effort should be placed on adequate hygiene since the universal strategy only marginally reduces the number of infected cattle compares with the safety-control strategy.

## Figures and Tables

**Table 1:** Parameter values for the bovine anaplasmosis model (12).

Parameter	Description	Value (1/day)	Reference(s)
Π_*J*_	Juvenile cattle recruitment rate	0.000299	[8]
Π_*A*_	Adult cattle recruitment rate	0.000274	[9]
Π_*T*_	Nymph recruitment rate	0.001609	[8]
*β*_*J*_	Cattle transmission probability	0.00048	[10]
*β*_*A*_	Cattle transmission probability	0.00048	[10]
*β*_*T*_	Ticks transmission probability	0.00048	[11]
*β*_*E*_	Devices transmission probability	0.15	[11]
*ϕ*_*T*_	Tick biting rate	0.60	[12]
*ϕ*_*E*_	Devices contact rate	0.0192	[9]
*μ*_*B*_	Cattle natural death rate	8.216 × 10^−07^	[10]
*μ*_*T*_	Ticks natural death rate	4.4082 × 10^−06^	[10]
*μ*_*E*_	Mechanical device decay rate	0.50	Assumed
*δ*_*B*_	Disease-induced mortality rate	0.39	[6]
*σ*_1_	Disease progression rate	0.031	[6]
*σ*_2_	Disease progression rate	0.0289	[6]
*σ*_3_	Disease progression rate	0.0270	[6]
*α*_*B*_	Juvenile cattle maturation rate	0.0037	[8]
*α*_*T*_	Nymph ticks maturation rate	0.0056	Assumed
*ε*_*E*_	Devices contamination rate	0.00061	[10]
*p*	Fraction in cattle	0.20	[11, 13]
*k*	Fraction in ticks	0.10	[8]

**Table 2:** Parameter values and sensitivity indices for each of the parameters for the bovine anaplasmosis model (12).

Parameter	Description	Value (1/day)	Sensitivity index
Π_*J*_	Juvenile cattle recruitment rate	0.000299	0.4799
Π_*A*_	Adult cattle recruitment rate	0.000274	0.4373
Π_*T*_	Nymph recruitment rate	0.001609	0.0276
*β*_*J*_	Cattle transmission probability	0.00048	0.0257
*β*_*A*_	Cattle transmission probability	0.00048	0.0019
*β*_*T*_	Ticks transmission probability	0.00048	0.0276
*β*_*E*_	Devices transmission probability	0.15	0.9448
*ϕ*_*T*_	Tick biting rate	0.60	0.0552
*ϕ*_*E*_	Devices contact rate	0.0192	0.9448
*μ*_*B*_	Cattle natural death rate	8.216 × 10^−07^	−1.8883
*μ*_*T*_	Ticks natural death rate	4.4082 × 10^−06^	−0.0552
*μ*_*E*_	Mechanical device decay rate	0.50	−0.9448
*δ*_*B*_	Disease-induced mortality rate	0.39	−0.8833
*σ*_1_	Disease progression rate	0.031	0.0027
*σ*_2_	Disease progression rate	0.0289	0.1855
*σ*_3_	Disease progression rate	0.0270	0.6977
*α*_*B*_	Juvenile cattle maturation rate	0.0037	−0.0041
*α*_*T*_	Nymph ticks maturation rate	0.0056	0
*ε*_*E*_	Devices contamination rate	0.00061	0.9448
*p*	Fraction in cattle	0.20	0.0126
*k*	Fraction in ticks	0.10	0.0276

**Table 3:** Initial conditions used for the simulations of model (12) for the three control strategies.

*S*_*J*_(0) = 195	*A*_*J*_(0) = 5	*C*_*J*_(0) = 0	
*S*_*A*_(0) = 333	*A*_*A*_(0) = 3	*C*_*A*_(0) = 0	*I*_*A*_(0) = 3
*S*_*TJ*_(0) = 82	*I*_*TA*_(0) = 5	*S*_*T*_ *A*(0) = 420	*I*_*TJ*_(0) = 5
*E*_*M*_(0) = 1			

**Table 4:** Simulation results of the cumulative number of new cases at *t* = 250 days for the juvenile and adult cattle using the bovine-culling strategy.

Cattle (culling-control)	No control	Low culling	Moderate culling	High culling
Juveniles	2.0 × 10^2^	41.5	41.0	40.1
Adults	3.4 × 10^2^	72.4	70.9	68.1

**Table 5:** Simulation results of the cumulative number of new cases at *t* = 250 days for the juvenile and adult cattle using the safety-control strategy.

Cattle (safety-control)	No control	Low strategy	Moderate strategy	High strategy
Juveniles	2.0 × 10^2^	41.5	30.1	25.8
Adults	3.4 × 10^2^	72.4	28.0	12.2

**Table 6:** Simulation results of the cumulative number of new cases at *t* = 250 days for the juvenile and adult cattle using the universal-control strategy.

Cattle (Universal strategy)	No Control	Low strategy	Moderate strategy	High strategy
Juveniles	2.00 × 10^2^	41.5	30.0	25.3
Adults	3.4 × 10^2^	72.5	27.4	11.6

## Appendix

### A. Proof of Lemma 1

See Lemma 1

Proof Let *t*_1_ = sup⁡{*t* > 0 : *F*(*t*) > 0 ∈ [0, *t*]}. Thus, *t*_1_ > 0. It follows from the first equation of the system (12), that

(A.1) $$\begin{array}{c} \frac{dS_{J}}{dt}=\Pi_{J}-\lambda_{J}S_{J}-\left(\;\alpha_{B}+\mu_{B}\;\right)S_{J} \end{array}$$

which can be rewritten as

(A.2) ddtSJtexp⁡∫0t1λJζdζ+k1t=ΠJexp⁡∫0t1λJζdζ+k1t,

where *k*_1_ = *α*_*B*_ + *μ*_*B*_. Hence,

(A.3) SJt1exp⁡∫0t1λJζdζ+k1t1−SJ0=∫0t1ΠJexp⁡∫0pλJζdζ+k1pdp

so that

(A.4) SJt1=SJ0exp⁡−∫0t1λJζdζ+k1t1+exp⁡−∫0t1λJζdζ+k1t1×∫0t1ΠJexp⁡∫0pλJζdζ+k1pdp>0.

Similarly, it can be shown that *F* > 0 for all *t* > 0. For the second part of the proof, note that 0 < *S*_*J*_(0) ≤ *N*_*B*_(*t*),  0 ≤ *A*_*J*_(0) ≤ *N*_*B*_(*t*),  0 ≤ *C*_*J*_(0) ≤ *N*_*B*_(*t*),  0 < *S*_*A*_(0) ≤ *N*_*B*_(*t*),  0 ≤ *A*_*A*_(0) ≤ *N*_*B*_(*t*),  0 ≤ *I*_*A*_(0) ≤ *N*_*B*_(*t*),  0 ≤ *C*_*A*_(0) ≤ *N*_*B*_(*t*),  0 < *S*_*TJ*_(0) ≤ *N*_*T*_(*t*),  0 ≤ *I*_*TJ*_(0) ≤ *N*_*T*_(*t*),  0 < *S*_*TA*_(0) ≤ *N*_*T*_(*t*),  0 ≤ *I*_*TA*_(0) ≤ *N*_*T*_(*t*),  0 ≤ *E*_*M*_(0) ≤ *N*_*T*_(*t*). Adding the cattle and tick component of the bovine anaplasmosis model (12) gives

(A.5) dNBtdt=ΠB−μBNBt−δBIBt,dNTdt=ΠT−μENT,dEMdt=εEAJ+CJ+AA+CA+IA−μEEM.

Hence,

(A.6) lim supt→∞ NBt≤ΠBμB,lim supt→∞ NTt≤ΠTμT,lim supt→∞ EMt≤εEΠBμEμB

as required.

### B. Proof of Lemma 2

See Lemma 2

Proof It follows from the sum of the first seven equations of model (12) that

(B.1) $$\begin{array}{c} \frac{dN_{B}\left(t\right)}{dt}=\Pi_{B}-\mu_{B}N_{B}\left(\;t\;\right)-\delta_{B}I_{A}\left(\;t\;\right), \end{array}$$

where Π_*B*_ = Π_*B*_ + 4Π_*A*_, so that

(B.2) $$\begin{array}{c} \frac{dN_{B}\left(t\right)}{dt}\leq \Pi_{B}-\mu_{B}N_{B}\left(\;t\;\right). \end{array}$$

Hence, *dN*_*B*_(*t*)/*dt* ≤ 0, if *N*_*B*_(0) ≥ Π_*B*_/*μ*_*B*_. Thus, *N*_*B*_(*t*) ≤ *N*_*B*_(0)*e*^−*μ*_*B*_*t*^ + (Π_*B*_/*μ*_*B*_)(1 − *e*^−*μ*_*B*_*t*^). In particular, *N*_*B*_(*t*) ≤ Π_*B*_/*μ*_*B*_. From the sum of the next four equations we have

(B.3) $$\begin{array}{c} \frac{dN_{T}}{dt}=\Pi_{T}-\mu_{E}N_{T}. \end{array}$$

Hence, *dN*_*T*_(*t*)/*dt* ≤ 0, if *N*_*T*_(0) ≥ Π_*T*_/*μ*_*T*_. Thus, *N*_*T*_(*t*) ≤ *N*_*T*_(0)*e*^−*μ*_*T*_*t*^ + (Π_*T*_/*μ*_*T*_)(1 − *e*^−*μ*_*T*_*t*^). In particular, *N*_*T*_(*t*) ≤ Π_*T*_/*μ*_*T*_. From the last equations of model (12), we have

(B.4) $$\begin{array}{c} \frac{dE_{M}\left(t\right)}{dt}=\varepsilon_{E}\left(\;A_{J}+C_{J}+A_{A}+C_{A}+I_{A}\;\right)-\mu_{E}E_{M}. \end{array}$$

Equation (B.2) gives

(B.5) $$\begin{array}{c} A_{J}\left(\;t\;\right)+C_{J}\left(\;t\;\right)+A_{A}\left(\;t\;\right)+C_{A}\left(\;t\;\right)+I_{A}\left(\;t\;\right)\leq \frac{\Pi_{B}}{\mu_{B}}. \end{array}$$

Hence, *dE*_*M*_/*dt* ≤ 0, if *E*_*M*_(0) ≥ *ε*_*E*_Π_*B*_/*μ*_*E*_*μ*_*B*_. Thus, *E*_*M*_(*t*) ≤ *E*_*M*_(0)*e*^−*μ*_*E*_*t*^ + (*ε*_*E*_Π_*B*_/*μ*_*E*_*μ*_*B*_)(1 − *e*^−*μ*_*E*_*t*^). In particular *E*_*M*_(*t*) ≤ *ε*_*E*_Π_*B*_/*μ*_*E*_*μ*_*B*_. Thus, the region *Ω* is positively invariant. Furthermore, if *N*_*B*_(0) > Π_*B*_/*μ*_*B*_, *N*_*T*_(0) > Π_*T*_/*μ*_*T*_ and *E*_*M*_(0) > *ε*_*E*_Π_*B*_/*μ*_*E*_*μ*_*B*_, then either the solutions enter *Ω* in finite time, or *N*_*B*_(*t*) approaches Π_*B*_/*μ*_*B*_, *N*_*T*_(*t*) approaches Π_*T*_/*μ*_*T*_, and *E*_*M*_(*t*) approaches *ε*_*E*_Π_*B*_/*μ*_*E*_*μ*_*B*_ asymptotically. Hence, the region *Ω* attracts all solutions in *ℝ*_+_^12^.

## Description of the Parameters and Variables for the Bovine Anaplasmosis Model (12)

### Variables

*S*_*J*_, *S*_*A*_: Population of susceptible juvenile and adult cattle
*A*_*J*_, *A*_*A*_: Population of infectious asymptomatic juvenile and adult cattle
*I*_*A*_: Population of infectious symptomatic adult cattle
*C*_*J*_, *C*_*A*_: Population of carrier juvenile and adult cattle
*S*_*TJ*_, *S*_*TA*_: Population of susceptible nymph (juvenile) and adult ticks
*I*_*TJ*_, *I*_*TA*_: Population of infectious nymph (juvenile) and adult ticks
*E*_*M*_: Population of infectious agent on mechanical device.

#### Parameters

Π_*J*_, Π_*A*_: Recruitment rate of juvenile and adult cattle
Π_*T*_: Recruitment rate of nymph ticks
*β*_*J*_, *β*_*A*_: Transmission probability per contact for susceptible cattle
*β*_*T*_: Transmission probability per contact for susceptible ticks
*β*_*E*_: Transmission probability per contact for mechanical device
*ϕ*_*T*_: Tick biting rate
*ϕ*_*E*_: Contact rate with the mechanical devices
*μ*_*B*_: Natural death rate of juvenile and adult cattle
*μ*_*T*_: Natural death rate of nymph and adult ticks
*μ*_*E*_: Decay rate of pathogen on mechanical device
*δ*_*B*_: Disease-induced mortality rate
*σ*_1_, *σ*_2_, *σ*_3_: Progression rate in cattle
*α*_*B*_: Maturation rate of juvenile cattle
*α*_*T*_: Maturation rate of nymph ticks
*ε*_*E*_: Mechanical device contamination rate
*p*: Fraction of susceptible adult cattle becoming symptomatic and asymptomatic
*k*: Fraction of recruited tick becoming susceptible and infectious.
